# Interdisciplinary Management of Concussion in an Adolescent With Type 1 Diabetes Mellitus: Diagnostic Challenges Related to Hypoglycemia and Cognitive Impairment

**DOI:** 10.7759/cureus.108045

**Published:** 2026-04-30

**Authors:** Chris Nelms, Carley C Whitt, Robert Kelly, William Ide, Sara Raiser

**Affiliations:** 1 Physical Medicine and Rehabilitation, University of Virginia, Charlottesville, USA

**Keywords:** concussion, hypoglycemia, interdisciplinary care, traumatic brain injury, type 1 diabetes mellitus

## Abstract

Type 1 diabetes mellitus (T1DM) can complicate recovery after mild traumatic brain injury (mTBI) by mimicking post-concussive symptoms. This case details the recovery of a 17-year-old female with T1DM who sustained an mTBI and lumbar spine fractures following a motor vehicle collision. Post-traumatic amnesia, short-term memory deficits, and impaired glucose regulation complicated her recovery. Cognitive symptoms of mTBI, such as working memory and attention deficits, can be worsened by nocturnal hypoglycemia, increasing the risk of poor glycemic control due to challenges with dietary and insulin management. Additionally, mTBI symptoms, such as headache and irritability, can overlap with hypoglycemia. At her one-week Physical Medicine and Rehabilitation (PM&R) follow-up, she reported persistent daily headaches, fatigue, neck pain, and nightly glucose readings in the 40s. In addition to standard concussion follow-up, the primary team coordinated a same-day evaluation by pediatric endocrinology, allowing real-time insulin adjustment in the clinic. With interdisciplinary management, the patient returned to her pre-injury cognitive baseline, her headaches improved, and her glucose levels stabilized. Misattributing symptoms to either mTBI or hypoglycemia can delay targeted treatment for both conditions, leading to worse functional outcomes and a prolonged rehabilitation course. This case highlights the importance of glycemic monitoring and team-based management in adolescents with T1DM recovering from concussion. It also illustrates the absence of standardized management strategies for adolescents with concurrent mTBI and T1DM.

## Introduction

A concussion is a subset of mild traumatic brain injury (mTBI) that results in impaired brain function secondary to biomechanical trauma but is not associated with loss of consciousness or structural changes on imaging [1-2]. Concussion commonly affects individuals involved in motor vehicle collisions, as well as those participating in high-impact sports. Most patients recover from concussions within one to two weeks, though some patients with comorbid conditions, such as migraines or metabolic disorders, may experience prolonged recovery [3]. Adolescents with type 1 diabetes mellitus (T1DM) face unique challenges during concussion recovery. Cognitive deficits secondary to concussion can compromise insulin administration, dietary management, and glucose monitoring. Hypoglycemia, in particular, may precipitate neurologic symptoms that are difficult to distinguish from post-concussion cognitive impairment. These symptoms include confusion, impaired attention, memory deficits, dizziness, blurry vision, and behavioral changes. Failure to identify their etiology poses a unique safety risk and delays appropriate intervention in this patient population [4]. In adolescents with T1DM, concussion presents a diagnostic challenge due to the potential for overlapping neurocognitive and hypoglycemic symptoms that can complicate management and delay recovery. Despite these difficulties, there is a lack of literature regarding the evaluation and management of concussion in individuals with T1DM.

## Case presentation

A 17-year-old, 49-kilogram female with a history of T1DM and migraine headaches presented to the ED following a motor vehicle collision, without a prior history of trauma. She was an unrestrained passenger in a rollover collision with unknown loss of consciousness and was amnestic to the event on initial examination. Prior to her injury, she had no reported academic difficulties or neurodevelopmental concerns, suggesting intact baseline cognitive function.

Her medical history was significant for newly diagnosed T1DM, managed with a basal-bolus insulin regimen, as well as a history of episodic migraines. She was bilingual, speaking fluent English and Spanish, but her parents spoke only Spanish. Her initial examination in the trauma bay revealed short-term memory deficits and delayed recall consistent with post-traumatic amnesia. Her cranial nerves were grossly intact, with normal pupil examination, intact sensation to light touch in the V1-3 distribution, symmetrical facial expression at rest and with activation, and fluent speech without dysarthria. Her strength was full, 5/5, on confrontation testing with bilateral hand grip, elbow flexion and extension, hip flexion, and ankle dorsiflexion and plantar flexion. Her sensation to light touch was intact in all extremities. Her initial head CT was also normal. The primary team consulted Pediatric Physical Medicine and Rehabilitation (PM&R) one day after her injury for concussion evaluation and management. She had cervical paraspinal muscle tenderness with an unchanged neurologic examination from her evaluation in the ED. A modified version of the Orientation Log (O-Log) evaluated her orientation to person, place, time, and circumstance following her mTBI, on which she scored 30 out of 30 possible points. This score indicated that she was fully oriented across all domains assessed [5]. Her primary concerns included headache, neck and back pain, fatigue, and subjective difficulty with memory and concentration. Physiatry ultimately diagnosed her with mTBI and used a translator to educate her and her parents on post-concussion care and recovery.

Before her accident, she was struggling with poorly controlled T1DM. Pediatric Endocrinology initiated inpatient glucose management and resumed her home insulin regimen with Humalog for carbohydrate coverage, 1 unit per 8 grams of carbohydrate; hyperglycemia correction, 1 unit per 40 mg/dL above a target blood glucose of 120 mg/dL; and urine ketone correction, 1 extra unit for moderate ketones and 2 extra units for large ketones, along with Lantus 15 units every 24 hours. They provided her with a continuous glucose monitor (CGM) and instructed her on how to use it before discharge. She was discharged home with instructions for brace wear, concussion precautions, glucose monitoring, and family supervision of insulin administration. Outpatient follow-up was arranged with endocrinology and the acquired brain injury (ABI) clinic through Pediatric Physiatry.

At a follow-up ABI Clinic visit, she reported ongoing headache, fatigue, intermittent tremor, neck and back pain, and recurrent overnight hypoglycemia, with CGM alarms registering values in the 40s-50s. During the clinic evaluation, her blood glucose was 65 mg/dL. She was promptly given orange juice and instructed to wait 15 minutes before rechecking her CGM. The clinical team emphasized that she would not be discharged from the clinic until her blood glucose was safely >70 mg/dL. After a subsequent check, her glucose rose to 71 mg/dL, thus addressing her acute hypoglycemia. She also reported improvement in her headache and tremors. Pediatric PM&R contacted her pediatric endocrinology care team, who sent a nurse to evaluate the patient in the Pediatric PM&R clinic and decreased her long-acting Lantus from 15 units daily to 14 units daily. No changes were made to her correctional insulin regimen. The nurse instructed her to eat a snack before bedtime to maintain glucose levels >100 mg/dL, emphasizing a carbohydrate-protein combination to reduce the risk of nocturnal hypoglycemia.

At the three-month physiatry follow-up, she demonstrated resolution of post-traumatic amnesia and subjective cognitive concerns, had returned to her full academic schedule, reported only infrequent fatigue-related headaches occurring once weekly, maintained stable glycemic control without recurrent hypoglycemia after insulin adjustment, exhibited no focal neurologic deficits on examination, and had resumed gym workouts without restriction (Table 1). Headaches were present but improved compared with her pre-injury baseline migraine history. She was referred to Neuropsychology to assess for subtle deficits relevant to diabetes management, and her blood glucose levels were more stable following endocrinology adjustment.

**Table 1:** Clinical and cognitive status at initial PM&R evaluation and at three-month follow-up. PM&R: Physical Medicine and Rehabilitation.

Parameter	Initial PM&R Evaluation (Post-Injury)	3-Month Follow-Up
Date relative to injury	10 days post-injury	95 days post-injury
Orientation (O-Log)	30/30	Not repeated; clinically normal on examination
Post-traumatic amnesia	Present; anterograde and retrograde	Resolved
Headache frequency	Daily, 3-8/10, triggered by noise	Once weekly when fatigued
Fatigue	Increased sleep, 12 AM-1 PM plus daytime naps	Sleeping from 12 AM to 6 AM without excessive daytime sleepiness
Cognitive concerns	Reported forgetfulness	Denied; returned to full academic schedule
Hypoglycemia	Overnight CGM readings in the 40s-50s	No recurrence after insulin adjustment
Diabetes management	Lantus decreased by 1 unit	No change in insulin regimen
Neurologic status	No focal deficits	No focal deficits
Activity level	Walking only	Returned to gym workouts

## Discussion

This case highlights the complex interplay between T1DM and post-concussion recovery. Both conditions can independently affect glucose metabolism, leading to overlapping symptoms of confusion, headache, irritability, and difficulty concentrating [6]. The International Society for Pediatric and Adolescent Diabetes (ISPAD) Guidelines emphasize the importance of glycemic control in adolescents for neurocognitive development, as severe hypoglycemic events can affect brain development, including cognition, language, and motor skills [7]. This adolescent with T1DM, whose post-concussive cognitive deficits were confounded by recurrent nocturnal hypoglycemia, required interdisciplinary care involving physiatry, endocrinology, orthopedics, and neuropsychology. Strengths include detailed longitudinal follow-up, clear documentation of overlapping metabolic and neurologic complications, and demonstration of successful interdisciplinary intervention. Limitations include limited generalizability from a single-patient case, potential unmeasured confounders, and reliance on self-reported adherence and symptoms.

Distinguishing post-concussive cognitive impairment from deficits secondary to impaired glycemic control is critical in adolescents with T1DM. Cognitive impairment is a common symptom of mTBI, and measures including attention, memory, processing speed, and reaction time are assessed in virtually every metric used to evaluate the neuropsychological health of athletes before and after a concussion [8]. Similarly, cognitive impairment is one of the key presenting symptoms of hypoglycemia, in addition to confusion, dizziness, blurred vision, memory deficits, and behavioral changes [9]. In a patient with T1DM who sustains a concussion, features that may help distinguish low blood sugar from concussion-related cognitive impairment include a temporal relationship between symptoms and low glucose readings, autonomic or neuroglycopenic signs such as sweating or tremor precipitated by missed meals or insulin-related factors, and symptom reversal with prompt glucose correction, as was seen in this patient [10].

Failure to rule out metabolic contributors to cognitive impairment in the post-concussive setting can lead to misattribution of persistent symptoms to concussion, resulting in inappropriate diagnosis and subsequent treatment of post-concussive syndrome. In a 2025 study, McIntosh SJ et al. concluded that cognitive symptoms, specifically difficulty concentrating, were associated with the greatest odds of persistent symptoms after concussion at one and six months; if these symptoms are inappropriately conflated with hypoglycemia, this could ultimately delay diabetes-specific interventions while increasing the risk of severe complications of hypoglycemia, such as permanent brain damage, seizures, coma, or death [11].

Adolescent diabetes management requires balancing emerging patient autonomy with appropriate caregiver oversight, particularly during neurologic recovery. Adolescence is characterized by a drive toward independent decision-making, which may conflict with the structured demands of diabetes management [12]. It is well documented that the transition from parental management to self-management of diabetes can present a challenge, especially when mTBI disrupts cognition, memory, and routines that support glycemic control [12]. Additionally, although the patient spoke English proficiently, her parents did not, which added a layer of complexity when educating the family on diabetes management. It is essential to use language interpreter services when parents do not speak the primary language, even when the adolescent appears fluent, as studies have shown that language discordance between healthcare providers and parents is associated with worse health outcomes [13]. This is particularly important to ensure adherence to concussion management and diabetes care plans, as communication gaps may compromise safety and recovery. Clinicians caring for this population should inquire about parents’ involvement in diabetes management and provide joint education on concussion and hypoglycemia symptoms and management. This approach helps ensure that communication gaps are addressed and that the adolescent’s neurologic recovery and metabolic comorbidities are managed appropriately.

This case identifies a gap in pediatric PM&R and rehabilitation literature, as there are currently no standardized protocols for managing patients with concurrent mTBI and T1DM in the adolescent population. Future research should focus on the development of standardized screening protocols to distinguish hypoglycemia from post-concussive symptoms in vulnerable populations, as well as interdisciplinary care pathways between physiatry and endocrinology. Additionally, prospective studies are needed to evaluate how glycemic variability impacts concussion recovery trajectories and functional outcomes.

## Conclusions

This case highlights how hypoglycemia can mimic or exacerbate concussion-related cognitive deficits in adolescents with T1DM, underscoring the need for interdisciplinary management and heightened diagnostic vigilance during neurologic recovery in this high-risk population. Adolescents with T1DM who sustain a concussion may experience overlapping cognitive deficits from both brain injury and hypoglycemia, complicating self-management of their chronic disease. Careful monitoring of glucose levels, early involvement of physiatry and endocrinology, and cross-specialty collaboration are critical to ensuring safe recovery and preventing secondary complications. Providers should maintain heightened awareness of subtle metabolic contributors to persistent cognitive symptoms and educate patients and families about the causes of cognitive impairment in the context of a patient’s comorbid conditions. Ultimately, providers must tailor interventions to address both the neurologic and endocrinologic needs of their patients.
